# Predicting glycated hemoglobin levels in the non-diabetic general population: Development and validation of the DIRECT-DETECT prediction model - a DIRECT study

**DOI:** 10.1371/journal.pone.0171816

**Published:** 2017-02-10

**Authors:** Simone P. Rauh, Martijn W. Heymans, Anitra D. M. Koopman, Giel Nijpels, Coen D. Stehouwer, Barbara Thorand, Wolfgang Rathmann, Christa Meisinger, Annette Peters, Tonia de las Heras Gala, Charlotte Glümer, Oluf Pedersen, Henna Cederberg, Johanna Kuusisto, Markku Laakso, Ewan R. Pearson, Paul W. Franks, Femke Rutters, Jacqueline M. Dekker

**Affiliations:** 1 Department of Epidemiology and Biostatistics and the EMGO Institute for Health and Care Research, VU University Medical Centre, Amsterdam, The Netherlands; 2 Department of General Practice and the EMGO Institute for Health and Care Research, VU University Medical Centre, Amsterdam, The Netherlands; 3 Department of Internal Medicine and Cardiovascular Research Institute Maastricht, Maastricht University Medical Centre, Maastricht, The Netherlands; 4 Institute of Epidemiology II, Helmholtz Zentrum München - German Research Center for Environmental Health, Neuherberg, Germany; 5 German Center for Diabetes Research (DZD), Neuherberg, Germany; 6 Institute of Biometrics and Epidemiology, German Diabetes Center, Leibniz Center for Diabetes Research at Heinrich Heine University Düsseldorf, Düsseldorf, Germany; 7 Research Centre for Prevention and Health, Glostrup Hospital, Capital Region of Denmark, Denmark; 8 The Novo Nordisk Foundation Center for Basic Metabolic Research, Faculty of Health and Medical Sciences, University of Copenhagen, Copenhagen, Denmark; 9 Department of Medicine, University of Kuopio and Kuopio University Hospital, Kuopio, Finland; 10 Medical Research Institute, Ninewells Hospital and Medical School, University of Dundee, Dundee, Scotland, United Kingdom; 11 Department of Clinical Sciences, Genetic and Molecular Epidemiology Unit, Lund University, Skane University Hospital Malmö, Malmö, Sweden; 12 Department of Public Health and Clinical Medicine, Umea University, Umea, Sweden; 13 Harvard Chan School of Public Health, Boston, Massachusetts, United States of America; Medizinische Universitat Innsbruck, AUSTRIA

## Abstract

**Aims/hypothesis:**

To develop a prediction model that can predict HbA1c levels after six years in the non-diabetic general population, including previously used readily available predictors.

**Methods:**

Data from 5,762 initially non-diabetic subjects from three population-based cohorts (Hoorn Study, Inter99, KORA S4/F4) were combined to predict HbA1c levels at six year follow-up. Using backward selection, age, BMI, waist circumference, use of anti-hypertensive medication, current smoking and parental history of diabetes remained in sex-specific linear regression models. To minimize overfitting of coefficients, we performed internal validation using bootstrapping techniques. Explained variance, discrimination and calibration were assessed using R^2^, classification tables (comparing highest/lowest 50% HbA1c levels) and calibration graphs. The model was externally validated in 2,765 non-diabetic subjects of the population-based cohort METSIM.

**Results:**

At baseline, mean HbA1c level was 5.6% (38 mmol/mol). After a mean follow-up of six years, mean HbA1c level was 5.7% (39 mmol/mol). Calibration graphs showed that predicted HbA1c levels were somewhat underestimated in the Inter99 cohort and overestimated in the Hoorn and KORA cohorts, indicating that the model’s intercept should be adjusted for each cohort to improve predictions. Sensitivity and specificity (95% CI) were 55.7% (53.9, 57.5) and 56.9% (55.1, 58.7) respectively, for women, and 54.6% (52.7, 56.5) and 54.3% (52.4, 56.2) for men. External validation showed similar performance in the METSIM cohort.

**Conclusions/interpretation:**

In the non-diabetic population, our DIRECT-DETECT prediction model, including readily available predictors, has a relatively low explained variance and moderate discriminative performance, but can help to distinguish between future highest and lowest HbA1c levels. Absolute HbA1c values are cohort-dependent.

## Introduction

Lifestyle and drug interventions can prevent or delay the development of type 2 diabetes in those at risk for the disease [1–3]. Therefore, it is important that screening tools are developed to identify those at risk. To facilitate the use in clinical practice, such a prediction model should include predictors that are non-invasive and should thus not include laboratory-based predictors [4]. In addition, non-invasive screening models can be useful for research purposes, for application in large databases where blood assays are not available. In the DIabetes REsearCh on patient straTification (DIRECT) study [5], there was a need for such a prediction model to select participants for a prospective cohort study.

Several non-invasive screening models have been developed to predict the risk of type 2 diabetes development [4]. One of those non-invasive prediction models that is often used is the Finnish diabetes risk score [6]. This risk score was developed as a simple screening tool predicting the risk of developing drug-treated type 2 diabetes within the next 10 years, using age, body mass index (BMI), waist circumference, use of anti-hypertensive drugs, and history of high blood glucose (such as gestational diabetes) as predictors [6]. In the Evaluation of Screening and Early Detection Strategies for Type 2 Diabetes and Impaired Glucose Tolerance (DETECT-2) project, an international data-pooling collaboration, the Finnish diabetes risk score was updated by including clinically diagnosed and screen-detected type 2 diabetes as endpoint, and by considering additional predictors: history of gestational diabetes, sex, smoking, and family history of diabetes [7]. Both the Finnish diabetes risk score and the DETECT-2 model showed adequate discrimination [6,7].

The diagnosis of type 2 diabetes in these models was however based on fasting glucose levels and/or glucose levels after an oral glucose tolerance test (OGTT). In 2010, glycated hemoglobin (HbA1c) levels have been added to the diagnostic criteria for diabetes [8]. HbA1c levels are strongly related to the risk of diabetic complications and show less variability compared to fasting glucose levels and 2h OGTT glucose levels [9]. Using Hba1c or glucose as criteria for T2D has been shown to identify additional and different amounts of diabetes patients [10,11]. However, to our knowledge, no non-invasive models have previously been developed to predict HbA1c levels in the non-diabetic population.

The aim of the current study was therefore to develop a prediction model that predicts HbA1c levels after six years in the non-diabetic population, including readily available predictors that are part of the DETECT-2 diabetes risk score [7]. We combined data from three European population-based cohorts to develop our DIRECT-DETECT prediction model and a fourth cohort to externally validate the model.

## Methods

### Study population

Three European population-based cohorts were used to develop the prediction model: the Hoorn Study [12], the Inter99 Study [13] and the Cooperative Health Research in the Region of Augsburg (KORA S4/F4 Study) [14].

- In the Hoorn Study (The Netherlands), 2,484 men and women aged 50 to 75 years participated at baseline (between 1989 and 1992). After 4–8 years, 1,513 of these participants had a follow-up examination [15]. At baseline and follow-up, HbA1c levels were determined by ion-exchange high-performance liquid chromatography (HPLC) [16], using a Modular Diabetes Monitoring System (Bio-Rad, Veenendaal, The Netherlands), with an inter-assay coefficient of variation of 3.3%.
- In the Inter99 Study (Denmark), 6,906 men and women aged 30 to 60 years participated at baseline (between 1999 and 2001). After 5–6 years, 4,031 of these participants had a follow-up examination [17]. At baseline, HbA1c levels were determined by HPLC (Bio-Rad, USA), with intra-assay and inter-assay coefficients of variation of < 1.5% and < 2%, respectively. Also at follow-up, HbA1c levels were determined by HPLC (TOSOH, Minato, Japan) [18], with intra-assay and inter-assay coefficients of variation of < 1% and < 2%, respectively.
- In the KORA S4/F4 Study (Germany), 1,653 men and women aged 55–74 years participated at baseline (between 1999 and 2001, called KORA S4). After 6–8 years, 1,209 of these participants had a follow-up examination, called KORA F4 [19]. At baseline, HbA1c levels were determined by a turbidimetric immunological method (Tina-Quant HBA1C II; Roche Diagnostics GmbH, Mannheim, Germany) on a Hitachi 717 instrument, with inter-assay coefficients of variation of 3.9% at HbA1c levels of 5.7% and 5.2% at HbA1c levels of 9.7%. At follow-up, HbA1c levels were determined with a reverse-phase cation-exchange high performance liquid chromatographic, photometric assay (A. Menarini Diagnostics, Florence, Italy) on a HA 8160 Hemoglobin Analysis System, with inter-assay coefficients of variation of 1.2% at HbA1c levels of 5.95% and 1.2% at HbA1c levels of 10.6%. To correct for assay differences between baseline and follow-up, baseline measures were transformed using a previously published method [20].

In these three cohorts (from now on referred to as ‘the development dataset’), participants with type 2 diabetes at baseline were excluded based on the following criteria: known diabetes, fasting plasma glucose levels ≥7.0 mmol/l, 2h OGTT glucose levels ≥11.1 mmol/l, and/or HbA1c levels ≥6.5% (48 mmol/mol; N = 673) [21]. After exclusion, information on HbA1c levels at baseline and follow-up and on the relevant predictors was available for 5,762 participants: N = 1,337 from the Hoorn study; N = 3,526 form the Inter99 Study and N = 899 from the KORA S4/F4 Study.

Additionally, the prediction model was externally validated in a fourth population-based cohort: the METSIM Study [22]. In the METSIM Study (Finland), 10,197 men aged 45–73 years participated at baseline (2005–2010) [23]. For the current study, 5-year follow-up data was available for 3,255 participants. Excluding participants with type 2 diabetes at baseline resulted in 2,765 eligible participants. At baseline and follow-up, HbA1c levels were determined with a Tosoh G7 glycohemoglobin analyser (Tosoh Bioscience, San Francisco, CA, USA) [24], with an inter-assay coefficient of variation of 2.8%.

Participants provided written informed consent. The Hoorn Study was approved by the VU University Medical Centre Ethics Committee. The Inter99 Study was approved by the Scientific Ethics Committee of the Capital Region of Denmark. The KORA S4/F4 Study was approved by the Ethics Committee of the Bavarian Medical Association. The METSIM Study was approved by the Ethics Committee of the University of Eastern Finland and Kuopio University Hospital. This work was undertaken as part of the DIabetes REsearCh on patient straTification (DIRECT) study, an EU FP7 Innovative Medicines Inititative (http://www.direct-diabetes.org/) that is described elsewhere [5].

### Data analysis

#### Developing the model predicting HbA1c levels at follow-up

A prediction model was developed considering non-invasive measures, which are part of the DETECT-2 risk score, as potential predictors: age, BMI, waist circumference, use of anti-hypertensive drugs (yes or no), smoking (current, former, or no) and parental history of diabetes (yes or no; for the KORA S4/F4 Study, the answer category ‘unknown’ was considered as ‘no’). As age, BMI and waist circumference showed no linear relationship with HbA1c levels at follow-up, these variables were categorized consistent with the DETECT-2 model [7]: age <45, ≥45 to <55, ≥55 to <65, ≥65 years; BMI <25, ≥25 to <30, ≥30 kg/m^2^; and waist circumference in sex-specific categories: <94, ≥94 to <102, ≥102 cm for men, and <80, ≥80 to <88, ≥88 cm for women. History of gestational diabetes was included in the original DETECT-2 risk score, but not in the current analysis, because information on this variable was available in only one of the datasets resulting in missing data for this variable for 77% of the women. For the same reason, information on family history of diabetes was limited to parental history, as information on diabetes history of siblings was missing for 27% of the participants. Sex-specific models were constructed.

Starting with the full model (i.e. including age, BMI, waist circumference, use of anti-hypertensive drugs, smoking and parental history of diabetes), we used a backward selection procedure to exclude variables that did not contribute significantly to the model. Significance was set at p<0.157 according to Akaike’s information criterion [25].

Additionally, we evaluated whether a correction was necessary for cohort by including a categorical variable for cohort source. As cohort source had an effect on the estimated regression coefficients, the regression coefficients of all predictors were first estimated including the cohort variable. Next, all regression coefficients were fixed and the cohort variable was removed from the model. Finally, using the fixed regression coefficients for all predictors (i.e. using the linear predictor of this model as an offset variable), a new cohort-independent intercept was calculated. This way, the regression coefficients for all predictors were corrected for the effect of cohort source without having a variable for cohort source in the model, making it possible to apply this model to new cohorts.

#### Internal and external validation

The prediction model was validated both internally and externally. Because prediction models typically perform better in the dataset that was used to develop the model compared to other datasets (owing to overfitting), validation is an important step after the development of a prediction model. Internal validation was performed using bootstrapping techniques: 500 bootstrap samples with replacement were taken from the original dataset. These bootstrap samples had the same size as the original dataset, and because the sampling included replacement, participants from the original dataset could appear in the bootstrap several times. In these bootstrap samples, the modelling process was repeated: regression coefficients were calculated and a backward selection was performed, resulting in a model for each bootstrap sample. These 'bootstrap models' were then applied to the original dataset. Next, the performance of the bootstrap models was evaluated both in the bootstrap sample and in the original dataset. For each bootstrap sample, the difference between the performance in the bootstrap sample and in the original dataset is called the *optimism*, which is a measure for model overfitting. The mean of these values is the optimism of the original prediction model [26,27]. Subsequently, the regression coefficients of our prediction model were adjusted for this optimism.

After this internal validation step, the optimism-corrected model was externally validated in the METSIM Study: the coefficients were applied to this external dataset and the external performance of the model was evaluated.

In the external dataset, no information was available on parental history of diabetes. However, information was available on family history, which was defined as either parents, siblings, or children with diabetes. To evaluate whether this difference could affect the performance of the model, we applied all regression coefficients of the prediction model to the METSIM Study, which included applying the parental history coefficient to the family history variable in the METSIM Study. Next, we applied all coefficients except the one for parental history to the METSIM Study and allowed the model to estimate the coefficient for family history. Finally, we compared the performance of these two models.

#### Performance

The performance of the prediction model was assessed in the development dataset and in the external validation dataset. We evaluated the explained variation of the models, which can be considered as an overall measure of the predictive ability of a model [26,28]. To assess calibration, i.e. the agreement between predicted and observed HbA1c levels, predicted HbA1c levels were divided in percentiles, and for each percentile the mean predicted and the mean observed HbA1c levels were displayed in a calibration graph.

To be able to evaluate the discriminative performance of the model, i.e. the ability of the model to discriminate between high and low HbA1c levels at follow-up, HbA1c levels were dichotomized. Within the Direct Study, the purpose of this prediction model was to select about half of the population of these existing cohort studies for inclusion in a new prospective study, and therefore, this dichotomization was performed using the median HbA1c level (HbA1c-levels < / ≥ 5.643% (38 mmol/mol) for men and < / ≥ 5.654% (38 mmol/mol) for women). Additionally, sensitivity and specificity were calculated, where sensitivity indicates the percentage of participants that were correctly classified as having high HbA1c levels among the total number of participants with high observed HbA1c levels, and specificity indicates the percentage of correctly classified participants among the participants with low observed HbA1c levels.

#### Sensitivity analysis

As a sensitivity analysis, we assessed the discriminative performance of the model when it is used to predict the incidence of pre-diabetes, defined as HbA1c levels ≥ 5.7% (mmol/mol) [21].

Secondly, we assessed the performance of the model when, next to the non-invasive predictors, baseline HbA1c levels were considered to predict HbA1c levels at follow-up.

### Software

Statistical analyses were performed using SPSS version 20 and R software version 2.15.2, using the packages ‘rms’ and ‘pROC’.

## Results

### Population characteristics

Table 1 shows the characteristics of the total development dataset and stratified per cohort: the Hoorn Study, the Inter99 Study, and the KORA S4/F4 Study. Furthermore, the table shows the characteristics of the external validation dataset: the METSIM Study. The mean HbA1c level at baseline was 5.6% (38 mmol/mol) in the development dataset and 5.7% (39 mmol/mol) in the external validation dataset. After a mean follow-up of 5.9 and 4.7 years, respectively, mean HbA1c levels were 5.7% (39 mmol/mol) in the development dataset and 5.8% (40 mmol/mol) in the external validation dataset.

**Table 1:** Baseline characteristics of the 4 datasets.

	Development datasets				External validation
	Total development dataset ^a^	Hoorn Study	KORA S4 Study	Inter99 Study	METSIM Study
N	5762	1337	899	3526	2765
Sex, % male	48%	46%	51%	48%	100%
Age, years	52.1 (10.5)	60.3 (6.8)	63.7 (5.4)	46.0 (7.6)	59.5 (5.8)
BMI, kg/m^2^	26.2 (4.0)	26.2 (3.1)	28.1 (4.0)	25.7 (4.1)	26.7 (3.5)
Waist circumference, cm					
• Men	93.5 (9.9)	94.0 (8.3)	99.7 (9.1)	91.6 (10.0)	97.1 (10.0)
• Women	82.0 (11.4)	85.7 (9.9)	89.1 (10.4)	78.7 (10.9)	NA
Use of antihypertensive drugs	12%	15%	32%	6%	38%
Current smoking	27%	30%	12%	30%	12%
Former smoking	31%	35%	36%	28%	44%
Parental history of diabetes	16%	14%	23%	15%	32% ^b^
Hba1c level at baseline, %	5.6 (0.4)	5.3 (0.4)	5.3 (0.4)	5.7 (0.4)	5.7 (0.3)
Hba1c level at baseline, mmol/mol	38 (4.4)	34 (4.4)	34 (4.4)	39 (4.4)	39 (3.3)
Hba1c level at follow-up, %	5.7 (0.4)	5.5 (0.6)	5.6 (0.4)	5.7 (0.3)	5.8 (0.4)
Hba1c level at follow-up, mmol/mol	39 (4.4)	37 (6.6)	38 (4.4)	39 (3.3)	40 (4.4)
*Follow-up*					
• Follow-up duration, years	5.9 (0.7)	6.4 (0.5)	7.1 (0.2)	5.4 (0.2)	4.7 (0.8)
• Cumulative incidence of type 2 diabetes, % ^c^	6%	11%	11%	3%	9%
• Incidence rate, n per 1,000 person years ^d^	10.8	17.9	16.4	5.8	25.5

Data are mean (SD) or % yes

BMI: body mass index

^a^ Total development dataset: Hoorn Study, KORA S4 Study and Inter99 Study combined

^b^ In the METSIM Study, no information was available on parental history of diabetes. Instead, information was available on family history (either parents, siblings, or children with diabetes)

^c^ Based on ADA 2014 criteria [21]

^d^ Estimated incidence rate in participants per 1,000 person-years was calculated by assumption that the date of diagnosis was in the middle of the follow-up period

Participants of the METSIM Study were on average older, had a larger waist circumference, used antihypertensive drugs more often, and smoked less often compared to the development dataset. Further, the prevalence of a positive family history in the external validation dataset was higher than the prevalence of a positive parental history of diabetes in the development dataset, and the external validation dataset only consisted of men (compared to 49% men in the development dataset).

### Predicting HbA1c levels at follow-up

For both men and women, age, BMI, waist circumference, use of anti-hypertensive medication, current smoking, and parental history of diabetes were significant predictors of HbA1c levels at follow-up. Table 2 shows the prediction model for men and women before and after correction for cohort source, and the final model after internal validation.

**Table 2:** Linear regression model predicting HbA1c levels after 6 years.

	Model without correction for cohort source, after backward-selection				Model corrected for cohort source, after backward-selection				Final model after internal validation	
	Women		Men		Women		Men		Women	Men
	Regression coefficient	P-value	Regression coefficient	P-value	Regression coefficient	P-value	Regression coefficient	P-value	Regression coefficient	Regression coefficient
Intercept	5.541	<0.001	5.621	<0.001	5.138	<0.001	5.231	<0.001	5.398	5.502
Age (years)										
• <45	[Reference]		[Reference]		[Reference]		[Reference]		[Reference]	[Reference]
• ≥45—<55	0.097	<0.001	-0.027	0.175	0.178	<0.001	0.055	0.005	0.173	0.053
• ≥55—<65	-0.010	0.665	-0.125	<0.001	0.219	<0.001	0.093	<0.001	0.213	0.091
• ≥65	-0.014	0.653	-0.113	<0.001	0.316	<0.001	0.193	<0.001	0.307	0.188
BMI (kg/m^2^)										
• <25	[Reference]		[Reference]		[Reference]		[Reference]		[Reference]	[Reference]
• ≥25—<30	0.041	0.083	-0.023	0.233	0.034	0.135	-0.029	0.123	0.033	-0.028
• ≥30	0.157	<0.001	0.064	0.070	0.111	0.001	0.033	0.326	0.108	0.032
Waist circumference (cm)										
• Female <80; male <94	[Reference]		[Reference]		[Reference]		[Reference]		[Reference]	[Reference]
• Female ≥80—<88; male ≥94—<102	-0.040	0.102	0.052	0.013	0.002	0.934	0.067	<0.001	0.002	0.065
• Female ≥88; male ≥102	0.043	0.152	0.116	<0.001	0.109	<0.001	0.118	<0.001	0.106	0.115
Use of anti-hypertensives (y/n)	0.055	0.034	0.059	0.023	0.051	0.042	0.052	0.039	0.050	0.050
Former smoking (y/n)										
Current smoking (y/n)	0.093	<0.001	0.125	<0.001	0.096	<0.001	0.137	<0.001	0.093	0.133
Parental history of diabetes (y/n)	0.074	<0.001	0.076	<0.001	0.073	<0.001	0.071	<0.001	0.071	0.069
Cohort source										
• Hoorn Study					[Reference]		[Reference]			
• KORA S4 Study					0.096	<0.001	0.164	<0.001		
• Inter99 Study					0.392	<0.001	0.390	<0.001		

BMI: body mass index

Explained variance of the final model after internal validation was 2% for women and 1.3% for men (Table 3). When assessed separately in the different cohorts, explained variance was 6.1%, 4.1%, and 14.7% for women and 7.1%, 9.7% and 5.2% for men from the Hoorn Study, KORA S4/F4 Study and Inter99 Study, respectively.

**Table 3:** Explained variance after internal validation.

	Women	Men
Total development dataset	2%	1.3%
• Hoorn Study	6.1%	7.1%
• KORA S4/F4 Study	4.1%	9.7%
• Inter99 Study	14.7%	5.2%
External validation: Metsim Study	4.3%	4.3%

**Fig 1:**
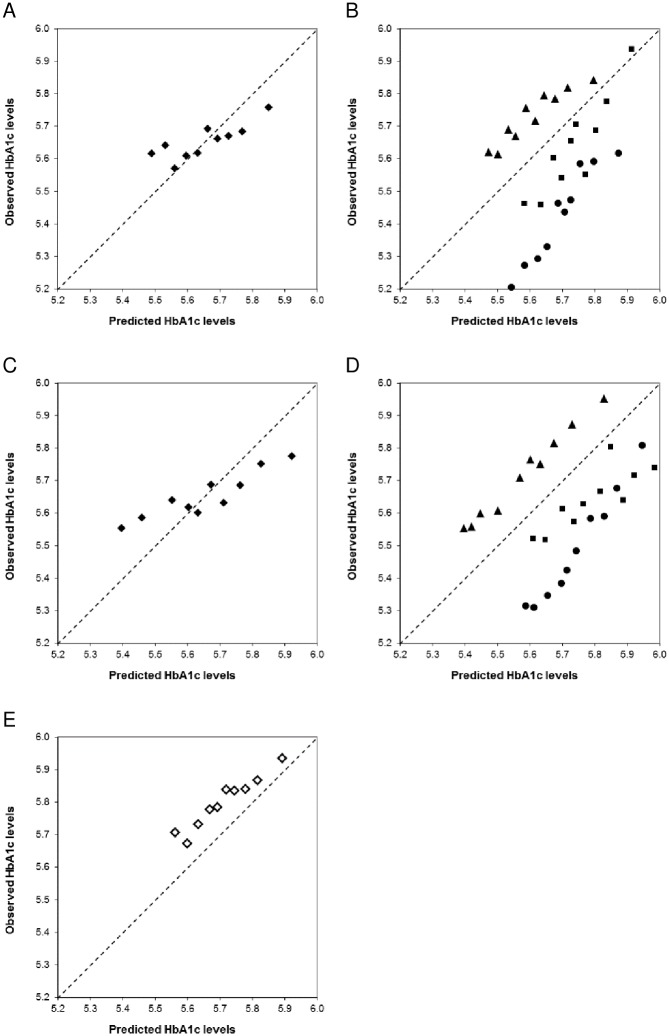
Calibration graphs (in deciles) of the prediction model after internal validation. (A) For men, in the development dataset (The Hoorn Study, The KORA S4/F4 Study and the Inter99 Study combined). (B) For men, in the development dataset, stratified per cohort: Hoorn Study (dots), KORA F4/S4 Study (squares), Inter99 Study (triangles). (C) For women, in the development dataset (The Hoorn Study, The KORA S4/F4 Study and the Inter99 Study combined). (D) For women, in the development dataset, stratified per cohort: Hoorn Study (dots), KORA F4/S4 Study (squares), Inter99 Study (triangles). (E) For men, in the external validation dataset (The METSIM Study). The diagonal line indicates perfect calibration.

Regarding calibration, the prediction model somewhat underestimated the lower observed HbA1c levels and somewhat overestimated the higher observed HbA1c levels, in both men (Fig 1A) and women (Fig 1C). Stratifying the calibration graphs for the different cohorts (Fig 1B and 1D) showed that the predictions were systematically overestimated in the Hoorn Study and the KORA S4/F4 Study, while they were systematically underestimated in the Inter99 Study. This indicates that absolute HbA1c levels were cohort specific, and updating the intercept for each cohort would improve calibration [26].

Table 4 shows the sensitivity and specificity of the prediction models for women and men when HbA1c levels were dichotomized using the median HbA1c level. Sensitivity (95% CI) was 55.7% (53.9, 57.5) for women and 54.6% (52.7, 56.5) for men, specificity (95% CI) was 56.9% (55.1, 58.7) and 54.3% (52.4, 56.2), respectively.

**Table 4:** Discriminative performance after internal validation ^a^.

*Discriminative performance in the development dataset* ^b^		
	Women	Men
Sensitivity (95% CI)	55.7% (53.9, 57.5)	54.6% (52.7, 56.5)
Specificity (95% CI)	56.9% (55.1, 58.7)	54.3% (52.4, 56.2)
*Discriminative performance in the external validation dataset* ^c^		
	Women	Men
Sensitivity (95% CI)	NA	56.4% (54.6, 58.2)
Specificity (95% CI)	NA	57.7% (55.9, 59.5)

^a^ To assess the discriminative performance of the model, HbA1c levels were dichotomized using the median HbA1c level (HbA1c-levels < / ≥ 5.643% (38 mmol/mol) for men and < / ≥ 5.654% (38 mmol/mol) for women).

^b^ Development dataset: Hoorn Study, KORA S4 Study and Inter99 Study combined

^c^ External validation dataset: METSIM Study

### External validation

External validation of the model showed an explained variance of 4.3% in the METSIM Study. The calibration graph (Fig 1E) indicates that the predictions were systematically underestimated in the METSIM Study.

In the external validation in the METSIM Study, sensitivity was 56.4% (95% CI 54.6, 58.2) and specificity was 57.7% (95% CI 55.9, 59.5), while 54.3% had observed ‘high’ HbA1c levels. Allowing for estimation of the regression coefficient for family history of diabetes did not considerably change the performance of the model, compared to applying the coefficient for parental history of diabetes to this variable (results not shown).

### Sensitivity analyses

When the model was used to predict the incidence of pre-diabetes, sensitivity (95% CI) was 55.6% (53.8, 57.4) for women and 36% (34.2, 37.8) for men and specificity (95% CI) was 65.3% (63.6, 67.0) and 71.3% (69.6, 73.0), respectively.

As expected, adding baseline HbA1c levels as a predictor next to the non-invasive predictors considerably improved model performance. Explained variance was 34% for women and 39% for men. Sensitivity (95% CI) of this model was 75.1% (73.6, 76.6) for women and 76.3% (74.7, 77.9) for men, and specificity (95% CI) was 72.2% (70.6, 73.8) and 74.6% (73.0, 76.2), respectively, when follow-up HbA1c levels were dichotomized using the median HbA1c level. When this model was used to predict the incidence of pre-diabetes, sensitivity (95% CI) was 69.3% (67.6, 71.0) and 71.4% (69.7, 73.1), and specificity (95% CI) was 79.1% (77.6, 80.6) and 78.8% (77.3, 80.3) for women and men, respectively.

## Discussion

Our aim was to develop a prediction model for HbA1c levels after six years of follow-up in a non-diabetic general population, using a sex-specific model and including readily available predictors that are part of the DETECT-2 diabetes risk score [7]. We showed that for men and women, age, BMI, waist circumference, use of anti-hypertensive medication, current smoking and parental history of diabetes were relevant predictors of HbA1c levels at follow-up, although these predictors could only explain 4–15% of the observed variance in HbA1c levels within each cohort. In addition, the discriminative performance of the DIRECT-DETECT prediction model was moderate. Calibration of the model could be improved by using different intercepts for each cohort.

Previous studies on non-invasive prediction models predicted the risk of developing type 2 diabetes, either drug-treated, clinically diagnosed, self-reported and/or screen-detected [6,7,29–38]. However, none of these models included HbA1c levels as a diagnostic criterion to define type 2 diabetes. We showed that non-invasive predictors can also be used to predict HbA1c levels after six years, although with moderate performance. Predictors for higher HbA1c levels in our study were also associated with a higher risk of developing type 2 diabetes in previous non-invasive models: higher age [6,7,29–32,35–37], higher waist circumference [6,7,29–31,33,37], use of anti-hypertensive medication [7,31,32,36,38], current smoking [7,30–32,36–38], and parental history of diabetes [7,29–31,35,36]. In line with previous studies, we observed higher HbA1c levels with a higher BMI [6,7,32,35,36]. Only for men, a small and not statistically significant negative regression coefficient was found for the middle BMI category. As the total BMI-variable did contribute to the model, it was kept in the model. Finally, previous prediction models that included former smoking had inconclusive results [7,32,36,37]. In our study, we included former smoking as a potential predictor, but this factor was excluded after backward selection.

A limitation of our study is that our development dataset included data from three cohorts with some population differences between the cohorts, and different assays were used to measure HbA1c levels. We corrected for these differences by correcting for cohort source. We observed that predicted HbA1c levels were systematically overestimated in the Hoorn Study and the KORA S4/F4 Study, and underestimated in the Inter99 Study and in the external validation in the METSIM Study. This indicates that calibration of the model could be improved by applying cohort-specific intercepts. We therefore advise to estimate a new intercept when applying this model to new populations. In addition, we evaluated whether differences in follow-up duration between participants affected the results, which they did not do. A second limitation is that the cohorts that were used in this study contain predominantly Caucasians. While racial differences are observed in HbA1c levels [39,40], future research might evaluate the performance of the prediction model for other ethnic groups. A third limitation is the possibility of attrition bias: in the cohort studies that we used to develop our prediction model, participants at follow-up were on average more healthy at baseline compared to those only participating at baseline [15,17]. This could have led to an underestimation of the association between predictors at baseline and HbA1c levels at follow-up. Finally, our external validation cohort, the METSIM Study, only consisted of men. This way, we were able to externally validate the prediction model for men, but not for women.

A strength of our study is that four large international population-based cohort studies were used to develop this prediction model, thus, results of this study are expected to be valid for Caucasian non-diabetic populations. Secondly, to our knowledge, we were the first to develop a non-invasive prediction model to predict HbA1c levels in the non-diabetic population.

The low explained variance and the moderate discriminative performance of the DIRECT-DETECT prediction model limit its use as a screening tool in clinical practice. Previous studies showed that additional information on blood lipid and glycaemic levels can improve the performance of a model [29]. In addition, our sensitivity analyses showed that adding baseline HbA1c levels to the prediction model, considerably improved model performance. Future research might therefore focus on developing a model including biomarkers that can predict change in HbA1c levels. However, the current prediction model can be used for purposes for which it was originally designed: as a first step in large databases where blood assays are not available, to select participants at risk of glycaemic deterioration for prevention or inclusion in clinical trials [5].

In conclusion, we found that non-invasive measurements—age, BMI, waist circumference, use of anti-hypertensive medication, current smoking and parental history of diabetes—were relevant predictors of HbA1c levels at follow-up both for men and women, although the explained variance and the discriminative performance of the model were moderate.
